# The inhibitory receptor Tim-3 fails to suppress IFN-γ production via the NFAT pathway in NK-cell, unlike that in CD4^+^ T cells

**DOI:** 10.1186/s12865-021-00417-9

**Published:** 2021-04-09

**Authors:** Xiaowen Yu, Bin Lang, Xi Chen, Yao Tian, Shi Qian, Zining Zhang, Yajing Fu, Junjie Xu, Xiaoxu Han, Haibo Ding, Yongjun Jiang

**Affiliations:** 1 NHC Key Laboratory of AIDS Immunology (China Medical University), National Clinical Research Center for Laboratory Medicine, The First Affiliated Hospital of China Medical University, No 155, Nanjing North Street, Heping District, Shenyang, 110001 Liaoning Province China; 2 Key Laboratory of AIDS Immunology, Chinese Academy of Medical Sciences, Shenyang, 110001 China; 3 Department of Clinical Laboratory, The Affiliated Hospital of Qingdao University, Qingdao, 266000 China; 4 Department of Clinical Laboratory, The Second Affiliated Hospital of Dalian Medical University, 467 Zhongshan Road, Dalian, 116023 China

**Keywords:** Tim-3, IFN-γ, NK cells, NFAT, HIV infection

## Abstract

**Background:**

T cell immunoglobulin and mucin domain-containing-3 (Tim-3) is a negative regulator expressed on T cells, and is also expressed on natural killer (NK) cells. The function of Tim-3 chiefly restricts IFNγ-production in T cells, however, the impact of Tim-3 on NK cell function has not been clearly elucidated.

**Results:**

In this study, we demonstrated down-regulation of Tim-3 expression on NK cells while Tim-3 is upregulated on CD4^+^ T cells during HIV infection. Functional assays indicated that Tim-3 mediates suppression of CD107a degranulation in NK cells and CD4^+^ T cells, while it fails to inhibit the production of IFN-γ by NK cells. Analyses of downstream pathways using an antibody to block Tim-3 function demonstrated that Tim-3 can inhibit ERK and NFκB p65 signaling; however, it failed to suppress the NFAT pathway. Further, we found that the NFAT activity in NK cells was much higher than that in CD4^+^ T cells, indicating that NFAT pathway is important for promotion of IFN-γ production by NK cells.

**Conclusions:**

Thus, our data show that the expression of Tim-3 on NK cells is insufficient to inhibit IFN-γ production. Collectively, our findings demonstrate a potential mechanism of Tim-3 regulation of NK cells and a target for HIV infection immunotherapy.

**Supplementary Information:**

The online version contains supplementary material available at 10.1186/s12865-021-00417-9.

## Introduction

Human immunodeficiency virus (HIV) infection can cause acquired immunodeficiency syndrome, which is characterized by CD4^+^ T cell depletion, recurrent opportunistic infections, and ultimately, immune exhaustion. During HIV infection, HIV-specific CD4^+^ T cells producing both interferon-gamma (IFN-γ) and IL-2 were associated with protective immunity [1, 2], and natural killer (NK) cells can secret IFN-γ to enhance antiviral reactions [3, 4]. In addition, NK cells can release perforin and granzymes from their cytoplasm after activation, indicated by lysosome-associated membrane protein-1 (LAMP-1, CD107a), a recognized marker of NK cell activity [5].

The function of NK cells is dependent on their balance of activating and inhibitory receptors, including c-type lectins, killer immunoglobulin receptors, natural cytotoxicity receptors, and FcγRIIIa receptor (CD16) [6]. Recently, T cell immunoglobulin and mucin-domain-containing molecule-3 (Tim-3) has also been reported to be expressed on NK cells as a receptor [7, 8], which was initially identified as an inhibitory receptor on terminally differentiated CD4^+^ T cells that suppresses cytokine production and promotes tumor proliferation, as well as HIV and hepatitis C virus infection [9–11].

Although Tim-3 has been reported to be expressed on NK cells, the specific details of its expression and function remain controversial. Jost et al. demonstrated reduced Tim-3 levels on NK cells from untreated individuals infected with HIV [12], while Finney et al. reported elevated Tim-3 levels on NK cells during HIV infection, particularly the CD56^bri^ subset [13]. It has been reported that Tim-3 is an inhibitory receptor that suppresses NK cell-mediated cytotoxicity in healthy subjects [14]; however, others reported that Tim-3 acts as an activating receptor, that is inducible by stimulation with various cytokines and functions to promote IFN-γ production [7]. Given this discordance in the findings of studies of the expression and function of Tim-3 on NK cells in HIV-infected individuals, further investigations are required to clarify the role of Tim-3 in NK cell function.

In this study, we found that Tim-3 expression is reduced on NK cells during HIV infection, while Tim-3 is upregulated on CD4^+^ T cells. Additionally, Tim-3 inhibits the expression of CD107a on NK cells and CD4^+^ T cells; however, we found that Tim-3 fails to inhibit IFN-γ production in NK cells, unlike in CD4^+^ T cells. Furthermore, we found that Tim-3 fails to inhibit the NFAT pathway, and that the NFAT pathway is important for induction of IFN-γ production in NK cells.

## Materials and methods

### Study subjects

Forty-one subjects were enrolled from the men who have sex with men (MSM) cohort of Voluntary Counseling and Testing for HIV and Red Ribbon clinics in the First Affiliated Hospital of China Medical University, including 13 HIV-negative controls (HIV^−^) and 28 subjects with chronic HIV infection (HIV^+^) who had been infected with HIV for > 1 year, and who had not received highly active antiretroviral therapy (HAART). The characteristics of the subjects enrolled in the study are presented in Table 1.

**Table 1:** Characteristics of subjects enrolled in this study

Characteristics	HAART-naïve HIV-infected subjects		HIV-negative controls
	CD4^**+**^ T cell high group	CD4^**+**^ T cell low group	
Number of subjects	15	13	13
Age (years); median (range)	25 (19, 51)	30 (25, 49)	26 (21, 44)
MSM (No, %)	15 (100%)	13 (100%)	13 (100%)
CD4^+^ T cell count (cells/μl); median (range)	560 (370–1229)	274 (139–347)	N/A
Viral load (copies/ml); median (range)	1380 (191–17,600)	68,900 (22900–226,000)	N/A
Time since infection (years); median (range)	2.54 (1–6.08)	2.28 (1.01–9.49)	N/A

*HAART* highly active antiretroviral therapy, *MSM* men who have sex with men

### Detection of Tim-3 expression

Cryopreserved peripheral blood mononuclear cells (PBMCs) were thawed and surface stained with CD3-FITC, CD4-APC-Cy7, CD56-PE-Cy7 (BD Biosciences), and Tim-3-PE (BioLegend). NK cells were defined as CD3^−^ CD56 ^+^ lymphocytes [12, 14]. All samples were acquired using an LSR II Fortessa cytometer (BD Biosciences), and data analyzed using FacsDiva™ 8.0.3 (URL: www.bdbiosciences.com) and FlowJo™ 10.5.0 (URL: www.flowjo.com/flowjo-eula/).

### IFN-γ and CD107a assays

PBMCs were thawed and stimulated with IL-12 (10 ng/mL, R&D) and IL-15 (50 ng/mL, R&D) in 96-well plates for 24 h at 37 °C in 5% CO_2_. CD107a-APC-Cy7 (BD Biosciences) and monensin (GolgiStop, BD Biosciences) were added into the wells 5 h before harvesting. Cells were harvested and washed with PBS, then surface stained with CD3-FITC, CD4-BV421, CD56-PE-Cy7, and Tim-3-PE. After fixing and permeabilizing with Fixation/Permeabilization Solution Kit (BD Biosciences), intracellular staining of anti-IFN-γ-APC (BD Biosciences) was conducted. Subsequently, cells were washed with PBS and fixed in 1% polyformaldehyde. The proportions of IFN-γ and CD107a-positive cells were detected using a BD LSR II and analyzed using FacsDiva™ 8.0.3 (URL: www.bdbiosciences.com) and FlowJo™ 10.5.0 (URL: www.flowjo.com/flowjo-eula/).

### Blockade using anti-Tim-3

PBMCs from HIV-negative donors were stimulated with IL-12 and IL-15 in 96-well plates (24 h, 37 °C, 5% CO_2_). Purified anti-human Tim-3 blocking antibody (20 μg/ml) or Purified mouse IgG_1_ κ isotype control antibody (BioLegend), CD107a-APC-Cy7, and monensin were added for 5 h before harvesting. Cells were harvested and washed with PBS, then surface stained with CD3-Percp-cy5.5, CD4-BV421, CD56-PE-Cy7, and Tim-3-PE. After fixing and permeabilizing, intracellular staining of IFN-γ-APC, Phospho-NFκB p65-PE, Alexa Fluor® 488 Mouse Anti-ERK1/2 (pT202/pY204), or Alexa Fluor® 488 anti-NFAT was conducted. Cells were then washed with PBS and analyzed by FlowJo™ 10.5.0 (URL: www.flowjo.com/flowjo-eula/).

### Detection of the effects of signaling pathway inhibitors

PBMCs from HIV-negative donors were stimulated with IL-12 and IL-15 in 96-well plates (24 h, 37 °C, 5% CO_2_). ERK inhibitor (PD98059, R&D), NFκB p65 inhibitor (PDTC, R&D), or NFAT inhibitor (480401-M, R&D) were added 6 h before harvesting. Cells were then harvested and analyzed by FlowJo™ 10.5.0 (URL: www.flowjo.com/flowjo-eula/).

### Reverse transcription and quantitative real-time PCR

Total RNA from PBMCs from HIV-negative donors was isolated using an RNeasy Plus Mini Kit (Qiagen) and reverse transcribed using a Primpscript® RT reagent kit (TAKARA, Japan), following the manufacturer’s protocol. Real-time PCR for detection of mRNA was performed using SYBR® Premix Ex Taq™ II (TAKARA), with the following primer sets (Beijing Genomics Institute, BGI): *IFN-γ* forward, 5′- CAG CTC TGC ATC GTT TTG GG and reverse, 5′- GTT CCA TTA TCC GCT ACA TCT GAA; and *GAPDH* forward, 5′- ACA TCG CTC AGA CAC CAT G and reverse, 5′- TGT AGT TGA GGT CAA TGA AGG G. mRNA expression levels were normalized to those of *GAPDH*. Changes in mRNA expression were calculated using the 2^−ΔΔCp^ method [15].

### Statistical analysis

The Mann-Whitney and Wilcoxon matched-pairs signed rank tests were used for comparisons of data between two groups. A *p*-value < 0.05 (two-tailed test) was considered statistically significant. All data analysis was performed using GraphPad Prism 9.0 (URL: www.graphpad.com).

## Results

### Tim-3 expression is decreased on NK cells but increased on CD4^+^ T cells in subjects with HIV infection

**Fig. 1:**
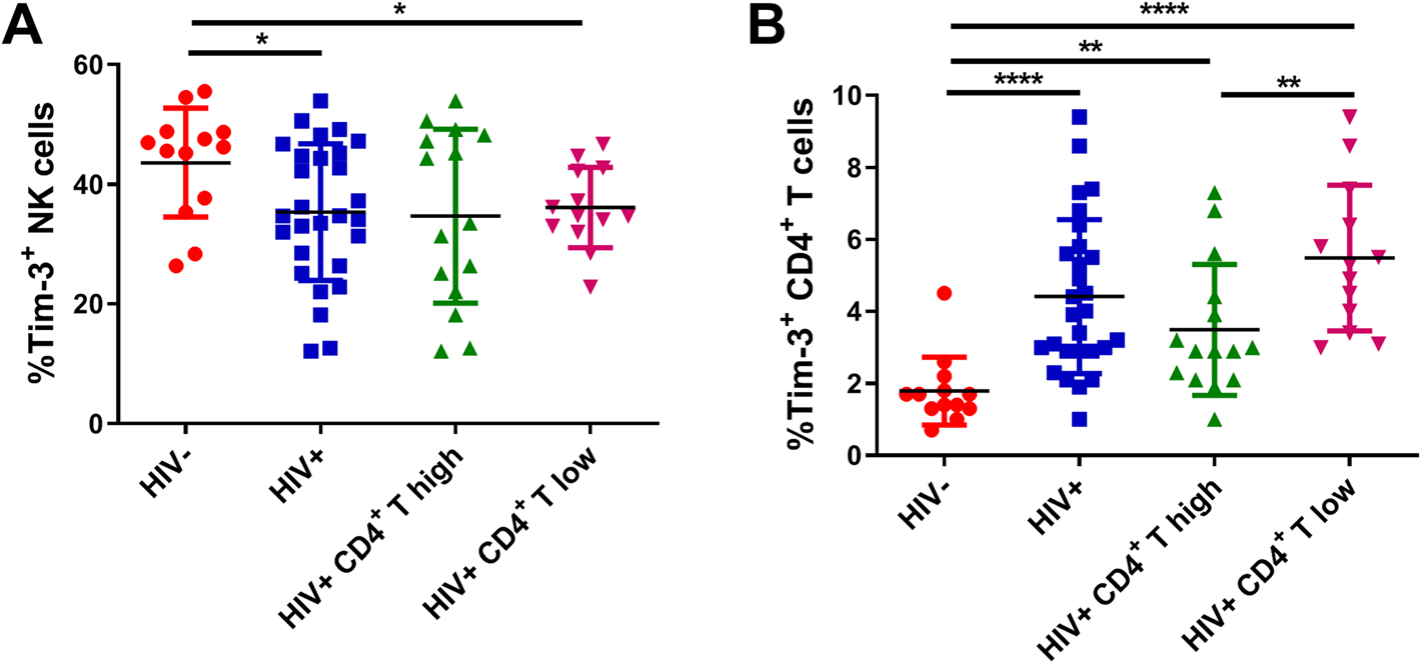
Tim-3 expression on NK cells and CD4^+^ T cells in HIV^−^, HIV^+^, HIV^+^ CD4^+^ T cell high, and HIV^+^ CD4^+^ T cell low groups. **a** The percentage of Tim-3 expression on NK cells (HIV^−^, *n* = 13; HIV^+^, *n* = 28; HIV^+^ CD4^+^ T cell high, *n* = 15; and HIV^+^ CD4^+^ T cell low, *n* = 13). **b** The percentage of Tim-3 expression on CD4^+^ T cells in each of the four groups (HIV^−^, *n* = 13; HIV^+^, *n* = 28; HIV^+^ CD4^+^ T cell high, *n* = 15; and HIV^+^ CD4^+^ T cell low *n* = 13). All data analysis was performed using GraphPad Prism 9.0 software (URL: www.graphpad.com). A Mann–Whitney test was used to compare groups; ^*^p < 0.05, ^**^p < 0.01, ^***^p < 0.001, ^****^p < 0.0001

Tim-3 expression was measured on NK cells and CD4^+^ T cells from HIV-negative controls (HIV^−^) and subjects with chronic HIV infection (HIV^+^). On NK cells, the percentage of cells expressing Tim-3 in both HIV^+^ subjects and the HIV^+^ CD4^+^ T cell low group were lower than that in HIV^−^ subjects (*p* = 0.0224 and 0.0159, respectively; Fig. 1a), while there was no significant difference between the HIV^+^ CD4^+^ T cell high group and HIV^−^ subjects. In contrast to the Tim-3 expression on NK cells, an increased percentage of Tim-3 expression was observed on CD4^+^ T cells from HIV^+^ subjects compared with HIV^−^ subjects (*p* < 0.0001), regardless of whether they had high (*p* = 0.0019) or low (*p* < 0.0001) CD4^+^ T cell counts, and the HIV^+^ CD4^+^ T cell low group had higher Tim-3 expression than that of the HIV^+^ CD4^+^ T cell high group (*p* = 0.0061; Fig. 1b). Our data suggest that Tim-3 expression is down-regulated on NK cells, but up-regulated on CD4^+^ T cells in subjects with HIV infection.

### There is no difference in IFN-γ production between Tim-3^+^ and Tim-3^−^ NK cells, unlike corresponding CD4^+^ T cells

**Fig. 2:**
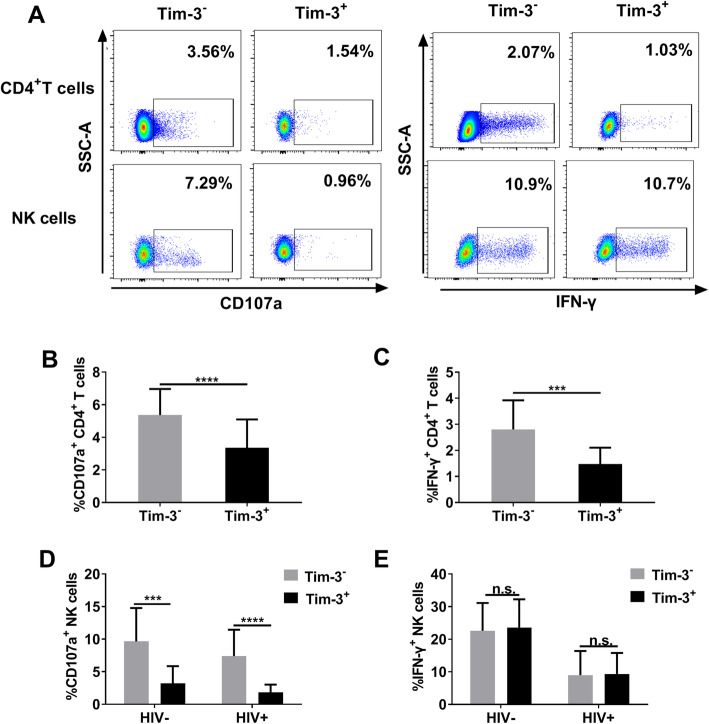
CD107a expression and IFN-γ production in Tim-3^−/+^ CD4^+^ T and Tim-3^−/+^ NK cells. **a** Representative flow cytometry plots of CD107a and IFN-γ levels in CD4^+^ T and NK cells. **b–c** CD107a expression and IFN-γ production in Tim-3^−/+^ CD4^+^ T cells from HIV^+^ subjects (*n* = 28). **d–e** CD107a expression and IFN-γ production in Tim-3^−/+^ NK cells in HIV^−^ (*n* = 13) and HIV^+^ (*n* = 28) subjects. All data analyzed using FacsDiva™ 8.0.3 (URL: www.bdbiosciences.com) and FlowJo™ 10.5.0 (URL: www.flowjo.com/flowjo-eula/). A Wilcoxon matched-pairs signed rank test (GraphPad Prism 9.0, URL: www.graphpad.com) was used to compare groups; ^*^p < 0.05, ^**^p < 0.01, ^***^p < 0.001, ^****^p < 0.0001

Following measurement of Tim-3 expression on NK and CD4^+^ T cells, we further evaluated differences in cell function between Tim-3^+^ and Tim-3^−^ NK or CD4^+^ T cells. Representative flow cytometry plots for CD107a expression and IFN-γ production in Tim-3^−/+^ CD4^+^ T cells and Tim-3^−/+^ NK cells are presented in Fig. 2a. Our data demonstrated that, in CD4^+^ T cells, the percentages of cells expressing CD107a and producing IFN-γ were lower in Tim-3^+^ populations than Tim-3^−^ populations in the HIV^+^ group (*p* < 0.0001 and *p* = 0.0002, respectively; Fig. 2b, c). Further, we found that CD107a expression on Tim-3^+^ NK cells was significantly decreased compared with Tim-3^−^ NK cells in both the HIV^−^ (*p* = 0.0002) and HIV^+^ groups (*p* < 0.0001; Fig. 2d); however, IFN-γ production did not differ significantly between Tim-3^+^ and Tim-3^−^ NK cells in either the HIV^−^ or HIV^+^ groups (Fig. 2e). Taken together, our results indicate that CD107a expression on Tim-3^+^ cells are decreased both on CD4^+^ T cells and NK cells. However, IFN-γ production in Tim-3^+^ NK cells is not reduced unlike in CD4^+^ T cells.

### Blockade of Tim-3 had no effect on IFN-γ production, but up-regulated CD107a expression on NK cells

**Fig. 3:**
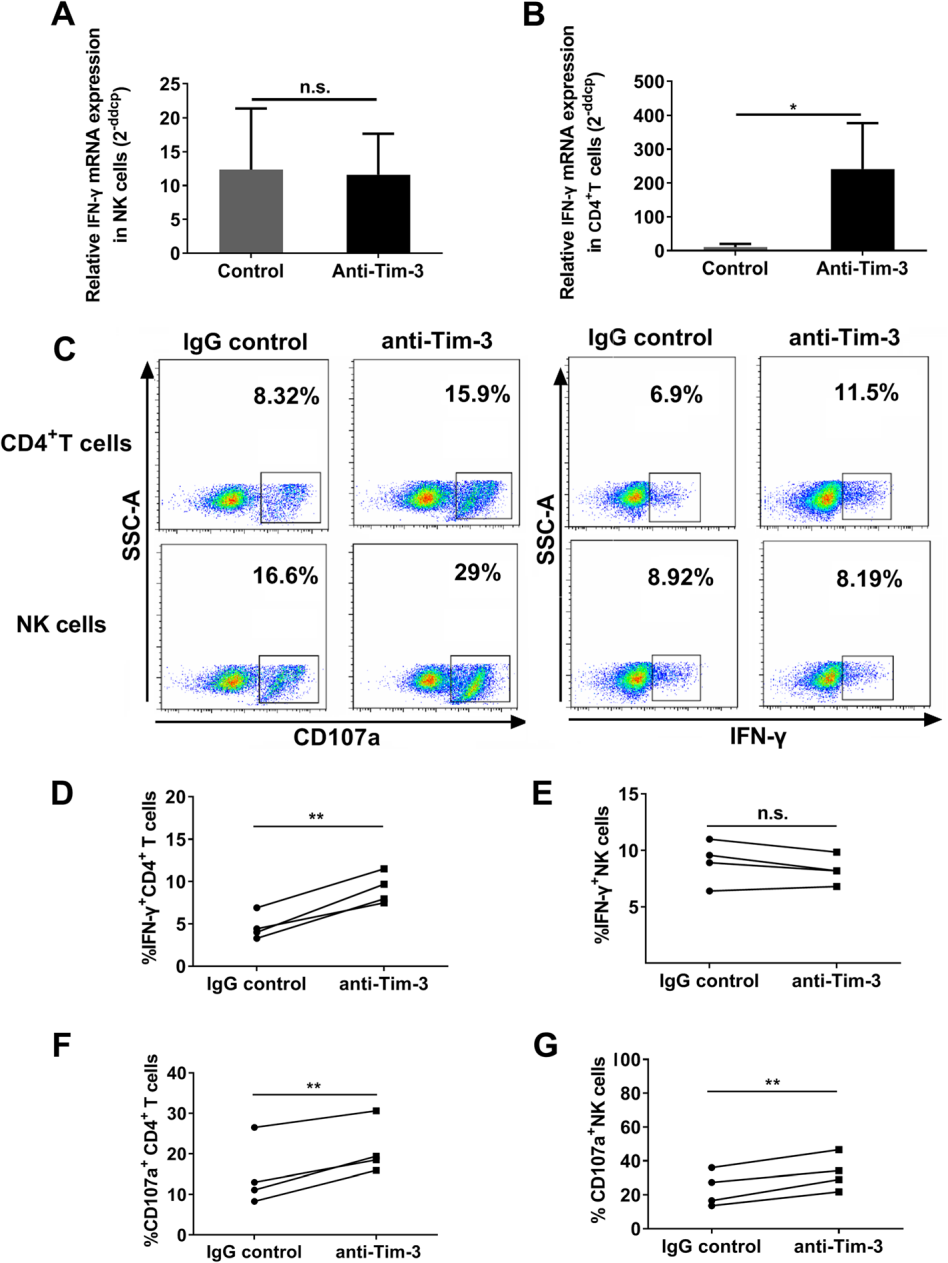
The influence of Tim-3 blockade on CD107a expression and IFN-γ production in CD4^+^ T and NK cells. **a–b** Relative mRNA expression of IFN-γ in NK and CD4^+ ^T cells with Tim-3 blockade (*n* = 4). **c** Representative flow cytometry plots of expression of CD107a expression and IFN-γ production in CD4^+^ T and NK cells incubated with anti-human Tim-3 blocking antibody or IgG control. **d–e** IFN-γ production in CD4^+^ T and NK cells with Tim-3 blockade (*n* = 4). **f–g** CD107a expression on CD4^+^ T and NK cells with Tim-3 blockade (*n* = 4). All data analyzed using FacsDiva™ 8.0.3 (URL: www.bdbiosciences.com) and FlowJo™ 10.5.0 (URL: www.flowjo.com/flowjo-eula/). A Wilcoxon matched-pairs signed rank test (GraphPad Prism 9.0, URL: www.graphpad.com) was used for comparisons between two groups; ^*^p < 0.05, ^**^p < 0.01, ^***^p < 0.001, ^****^p < 0.0001

Given the differences in CD107a expression and IFN-γ production we observed between Tim-3^−^ and Tim-3^+^ cells, we further explored whether Tim-3 expression had any effect on cell function. PBMCs from HIV-negative donors were stimulated with IL-12/IL-15 and incubated with Tim-3 blocking antibody. The cell viability after 24-h stimulation is still good, around 96.3% (supplementary Figure 1). Our data show that IFN-γ mRNA expression was not increased in NK cells (Fig. 3a) when Tim-3 was blocked, while it was upregulated in CD4^+^ T cells (*p* = 0.0286; Fig. 3b). Subsequently, the level of IFN-γ protein was detected by flow cytometry. Representative flow cytometry plots are presented in Fig. 3c. In CD4^+^ T cells, our data show that IFN-γ production was upregulated by Tim-3 blockade (*p* = 0.0036; Fig. 3d); however, this treatment failed to enhance IFN-γ production by NK cells (Fig. 3e), consistent with our mRNA results (Fig. 3a, b). CD107a expression on Tim-3+ and NK cells was also detected by flow cytometry. Representative flow cytometry plots are presented in Fig. 3c. We found that CD107a expression was upregulated on CD4^+^ T cells (*p* = 0.007; Fig. 3f) and NK cells (*p* = 0.0047; Fig. 3g) on application of the Tim-3 blocking antibody. Collectively, these data demonstrate that Tim-3 fails to suppress IFN-γ production by NK cells, despite acting as an inhibitory receptor on both NK and CD4^+^ T cells.

### Tim-3^+^ NK cells have reduced ERK and NFκB p65 phosphorylation compared with Tim-3^−^ NK cells, but have higher NFAT activity than Tim-3^+^ CD4^+^ T cells

**Fig. 4:**
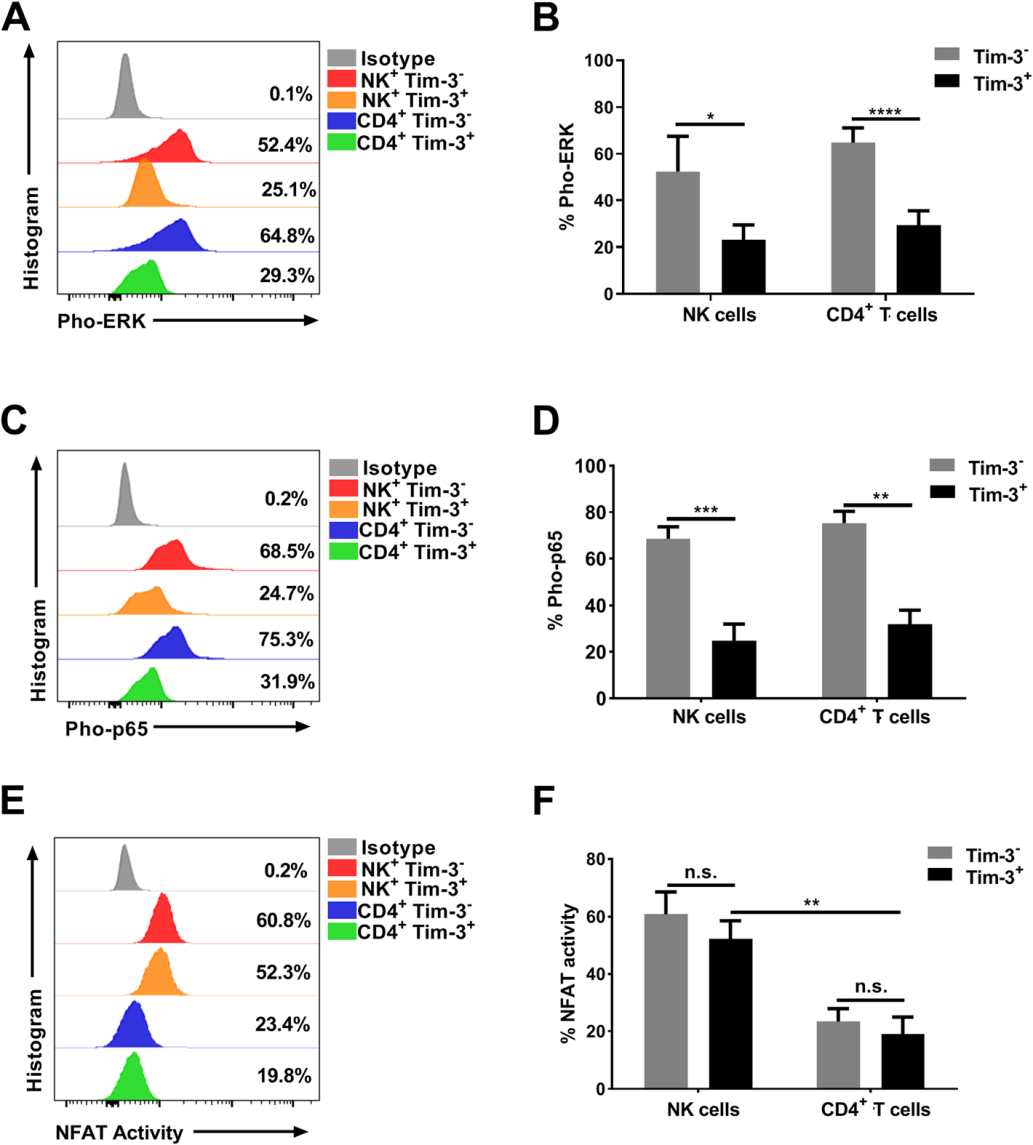
ERK phosphorylation, NFκB p65 phosphorylation, and NFAT activity in Tim-3^−/+^ NK and Tim-3^−/+^ CD4^+^ T cells. **a–b** Representative flow cytometry plots and statistical analyses of ERK phosphorylation in Tim-3^−/+^ NK and Tim-3^−/+^ CD4^+^ T cells (*n* = 4). **c–d** Representative flow cytometry plots and statistical analyses of NFκB p65 phosphorylation in Tim-3^−/+^ NK and Tim-3^−/+^ CD4^+^ T cells (*n* = 4). **e–f** Representative flow cytometry plots and statistical analyses of NFAT activity in Tim-3^−/+^ NK and Tim-3^−/+^ CD4^+^ T cells (*n* = 4). All data analyzed using FacsDiva™ 8.0.3 (URL: www.bdbiosciences.com) and FlowJo™ 10.5.0 (URL: www.flowjo.com/flowjo-eula/). Wilcoxon matched-pairs signed rank tests (GraphPad Prism 9.0, URL: www.graphpad.com) were used to compare the two groups; ^*^p < 0.05, ^**^p < 0.01, ^***^p < 0.001, ^****^p < 0.0001

IFN-γ production can be mediated by multiple pathways in both NK and CD4^+^ T cells, primarily the ERK [16], NFκB p65 [17], and NFAT [18, 19] signaling pathways. Based on our observation that Tim-3 is an inhibitory receptor, we further explored whether Tim-3 could also suppress these pathways. ERK and NFκB p65 phosphorylation and NFAT activity were analyzed in Tim-3^−/+^ NK cells and Tim-3^−/+^ CD4^+^ T cells. We found that the level of ERK phosphorylation in Tim-3^+^ NK cells was lower than that in Tim-3^−^ NK cells (*p* = 0.0322; Fig. 4a, b), with similar results in CD4^+^ T cells (*p* < 0.0001; Fig. 4a, b). Meanwhile, the much lower levels of NFκB p65 phosphorylation were detected in Tim-3^+^ NK cells and Tim-3^+^ CD4^+^ T cells (*p* = 0.0007 and *p* = 0.0043, respectively; Fig. 4c, d). Nevertheless, we found no significant difference in NFAT activity between Tim-3^+^ and Tim-3^−^ NK cells or between Tim-3^+^ and Tim-3^−^ CD4^+^ T cells. Further, we found that NFAT activity was much higher in Tim-3^+^ NK cells than that in Tim-3^+^ CD4^+^ T cells (*p* = 0.0076; Fig. 4e, f).

### Tim-3 blockade enhances ERK and NFκB p65 phosphorylation, but fails to alter NFAT activity in NK cells

**Fig. 5:**
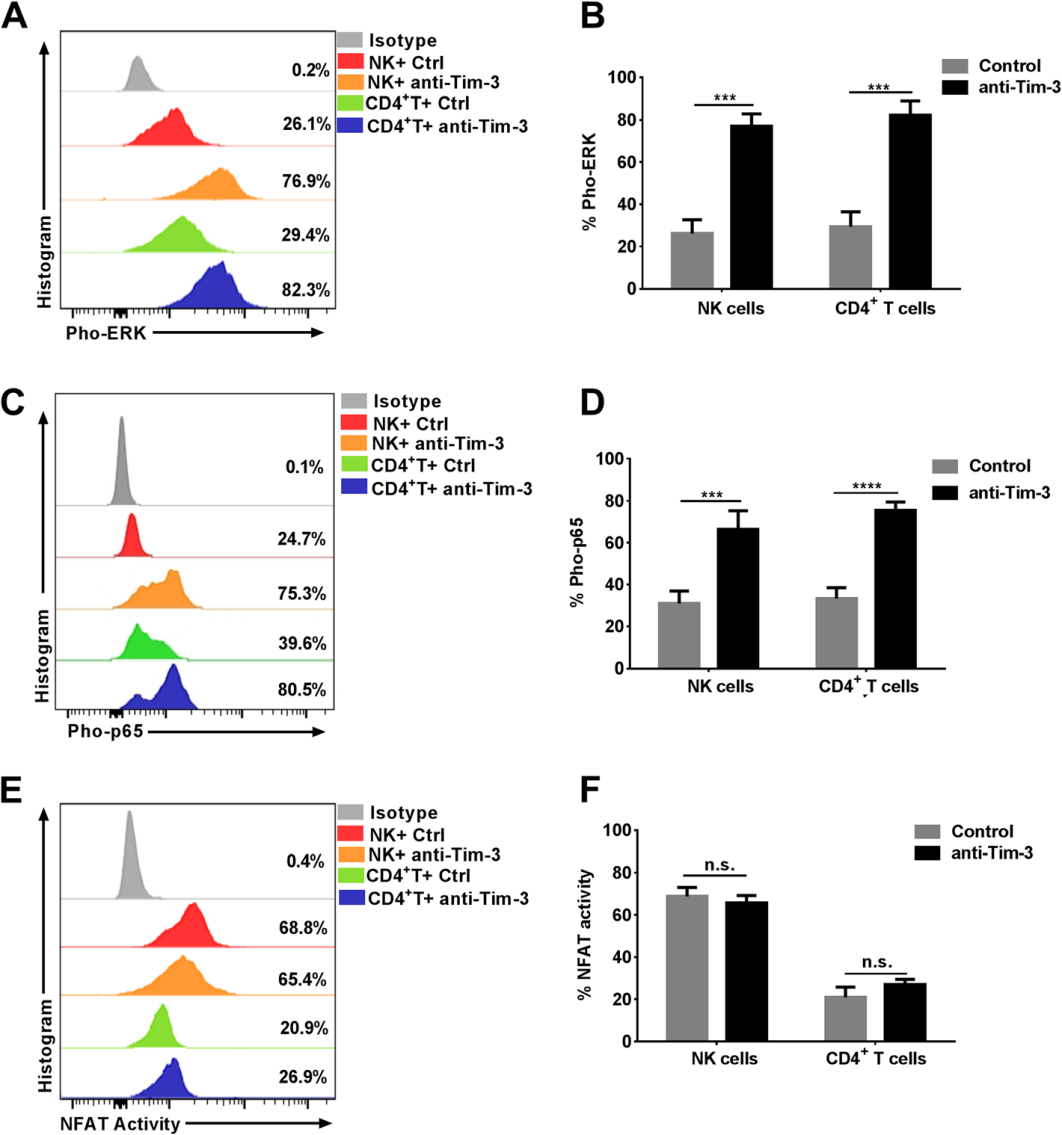
ERK phosphorylation, NFκB p65 phosphorylation, and NFAT activity following Tim-3 blockade in NK and CD4^+^ T cells. **a–b** Representative flow cytometry plots and statistical analyses of ERK phosphorylation following Tim-3 blockade in NK and CD4^+^ T cells (*n* = 4). **c–d** Representative flow cytometry plots and statistical analyses of NFκB p65 phosphorylation following Tim-3 blockade in NK and CD4^+^ T cells (*n* = 4). **e–f** Representative flow cytometry plots and statistical analyses of NFAT activity with Tim-3 blockade in NK and CD4^+^ T cells (*n* = 4). All data analyzed using FacsDiva™ 8.0.3 (URL: www.bdbiosciences.com) and FlowJo™ 10.5.0 (URL: www.flowjo.com/flowjo-eula/). Wilcoxon matched-pairs signed rank tests (GraphPad Prism 9.0, URL: www.graphpad.com) were used to compare the two groups; ^*^p < 0.05, ^**^p < 0.01, ^***^p < 0.001, ^****^p < 0.0001

Next, we investigated whether Tim-3 blockade had any effect on the ERK, NFκB p65, and NFAT pathways. As illustrated in Fig. 5a, b, levels of ERK phosphorylation in NK and CD4^+^ T cells were up-regulated after treatment with the Tim-3 blocking antibody (*p* = 0.0002 and *p* = 0.0003, respectively). In addition, we found that NFκB p65 phosphorylation levels were up-regulated in NK and CD4^+^ T cells on Tim-3 blockade (*p* = 0.0005 and *p* < 0.0001, respectively; Fig. 5c, d). Further, our data demonstrate that NFAT activity did not increase after blocking Tim-3 in NK and CD4^+^ T cells (Fig. 5e, f). Overall, these results indicate that Tim-3 suppresses ERK and NFκB p65 phosphorylation, but fails to inhibit NFAT activity in NK cells.

### NFAT signaling in NK cells has a more significant role in IFN-γ production than that in CD4^+^ T cells

**Fig. 6:**
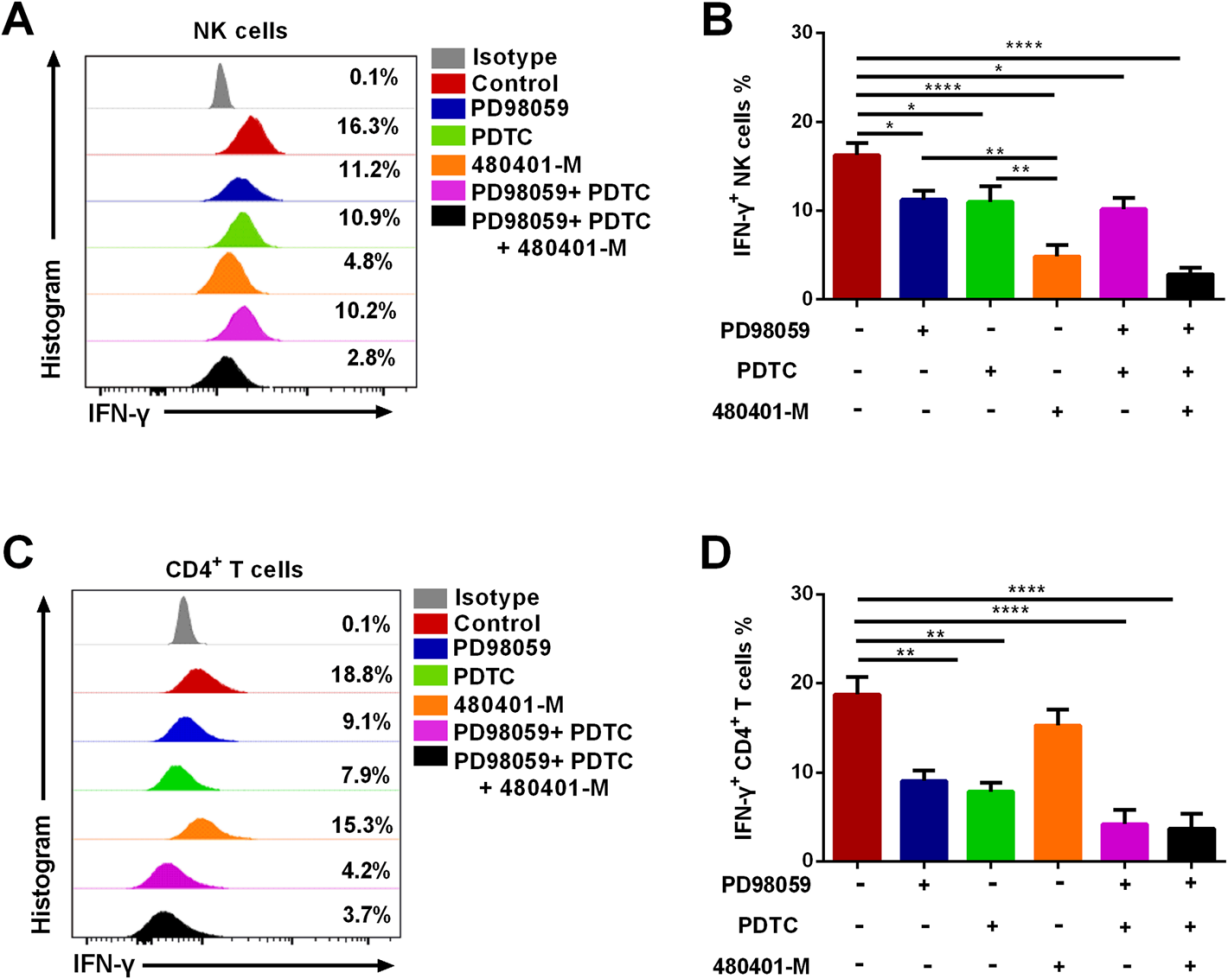
Influence of ERK, NFκB p65, and NFAT inhibitors on IFN-γ production by NK and CD4^+^ T cells. **a–b** Representative flow cytometry plots and statistical analyses of IFN-γ production following treatment with PD98059 (ERK inhibitor), PDTC (NFκB p65 inhibitor), 480401-M (NFAT inhibitor), PD98059 + PDTC, or PD98059 + PDTC + 480401-M, in NK cells (*n* = 4). **c–d** Representative flow cytometry plots and statistical analyses of IFN-γ production following PD98059, PDTC, 480401-M, PD98059 + PDTC, or PD98059 + PDTC + 480401-M treatment in CD4^+^ T cells (*n* = 4). All data analyzed using FacsDiva™ 8.0.3 (URL: www.bdbiosciences.com) and FlowJo™ 10.5.0 (URL: www.flowjo.com/flowjo-eula/). Wilcoxon matched-pairs signed rank tests (GraphPad Prism 9.0, URL: www.graphpad.com) were used to compare the two groups; ^*^p < 0.05, ^**^p < 0.01, ^***^p < 0.001, ^****^p < 0.0001

Next, we examined the contributions of the ERK, NFκB p65, and NFAT pathways to IFN-γ production by NK and CD4^+^ T cells, using the pathway inhibitors PD98059, PDTC, and 480401-M. Our results show that inhibition of the ERK, NFκB p65, or NFAT pathway could inhibit IFN-γ production (*p* = 0.0286, *p* = 0.0286, and *p* < 0.0001, respectively; Fig. 6a, b), and that 480401-M, the inhibitor of the NFAT pathways, more strongly suppressed IFN-γ, compared with PD98059 and PDTC (*p* = 0.0019 and *p* = 0.0052, respectively; Fig. 6a, b), indicating an important role for the NFAT pathway in promoting IFN-γ production by NK cells relative to ERK and NFκB p65 signaling; however, unlike NK cells, IFN-γ production in CD4^+^ T cells was not significantly decreased by inhibition of the NFAT pathway (Fig. 6c, d). Thus, our results suggest that the NFAT pathway has a significant role in IFN-γ production in NK cells, but not in CD4^+^ T cells.

## Discussion

Our study showed that Tim-3 expression was decreased on total NK cells in subjects with HIV infection, which is consistent with previous reports from Jost et al. [12] and de Kivit et al. [20]. One possible reason for the down-regulated Tim-3 expression on NK cells is high expression of its ligand, combined with Tim-3, leading to endocytosis and failure of cell surface detection; alternatively, the reduced expression might be due to feedback regulation of Tim-3 expression to promote NK cell cytotoxicity. Finney et al. reported that Tim-3 expression was elevated on NK cells during HIV infection [13]. The reason for our contrasting conclusions could be attributable to differences in the disease stage of the HIV-infected subjects in the two studies. Alternatively, there may be a difference in the study populations of the two studies, since the subjects with HIV infection in our study were MSM, with HIV^−^ MSM as controls. In studies of Tim-3 function, Ndhlovu et al. reported that, in samples from healthy donors, Tim-3 could inhibit NK cell-mediated cytotoxicity; however, they did not present data on IFN-γ production [14]. Further, Ju et al. demonstrated that stimulation with Tim-3 antibody could inhibit IFN-γ production by NK cells during HBV infection [21]; however, Gleason et al. proved that Tim-3 could enhance IFN-γ production as an activating receptor on NK cells, but did not investigate the possibility of NK cell cytotoxicity regulation by Tim-3 [7]. Evaluation of both IFN-γ production and CD107a expression represents a comprehensive approach to assessment of Tim-3 function in NK cells; therefore, we assayed both of these processes and our data clearly demonstrate that Tim-3 inhibits CD107a degranulation, but could not suppress IFN-γ production by NK cells.

**Fig. 7:**
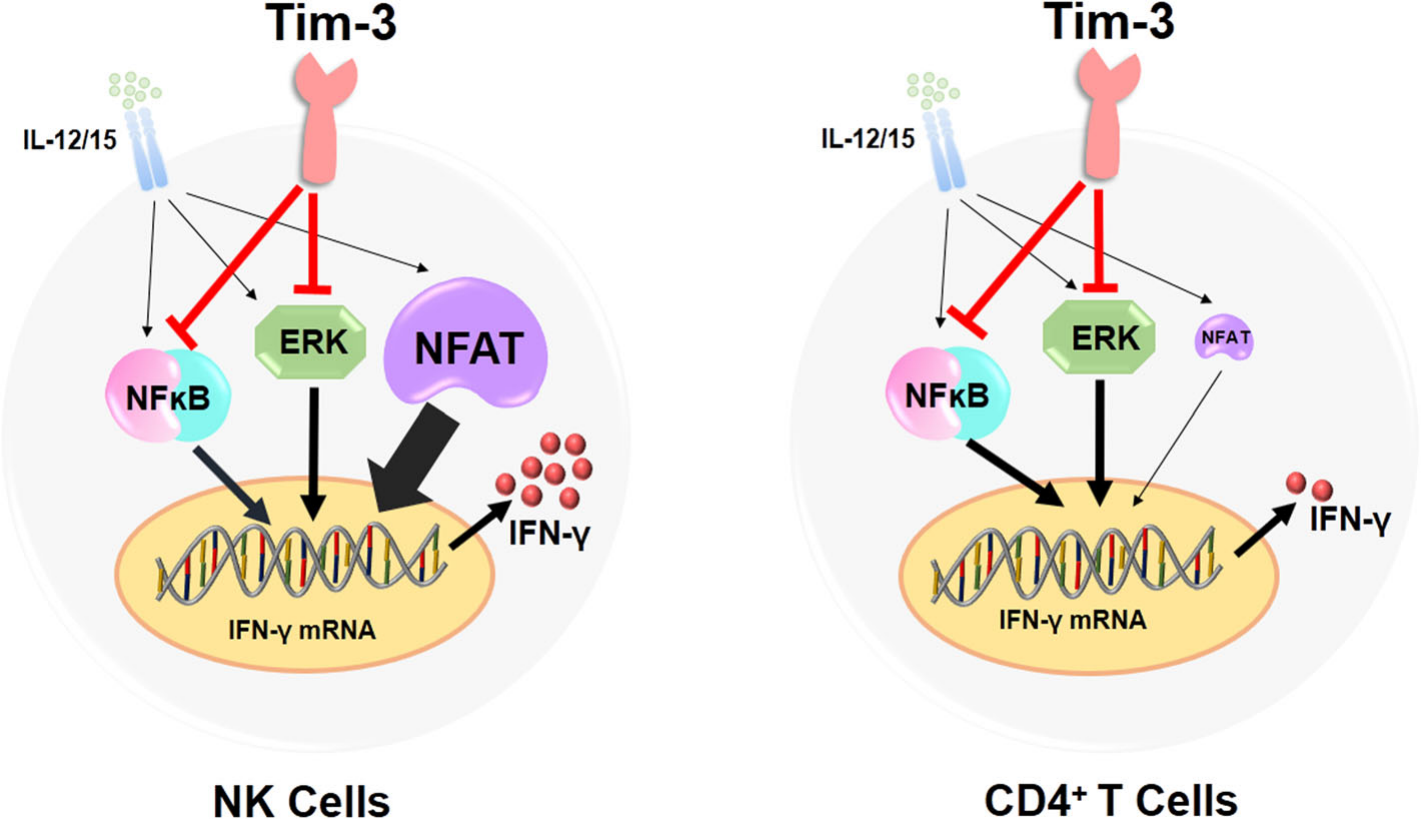
The mechanism of the effect of Tim-3 on IFN-γ production by NK and CD4^+^ T cells. Tim-3 can inhibit ERK and NFκB p65 signaling; however, it fails to inhibit the NFAT pathway, which is a major pathway promoting IFN-γ production by NK cells. Thus the production of IFN-γ in NK cells can be maintained via the NFAT pathway

Moreover, we further explored the signaling pathway mechanism that Tim-3 could not inhibit the production of IFN-γ on NK cells. The ERK [22], NFκB p65 [23], and NFAT [24, 25] pathways are important for IFN-γ production in NK cells. Gleason et al. reported that, as an activating receptor, Tim-3, could enhance IFN-γ production via ERK phosphorylation in NK cells [7]; however, contrary to those results, we found that the level of ERK phosphorylation in Tim-3^+^ NK cells was decreased relative to Tim-3^−^ NK cells, and that Tim-3 blockade could facilitate ERK phosphorylation, confirming that Tim-3 can act as an inhibitory receptor on NK cells to suppress ERK phosphorylation. In addition, we found that NFκB p65 phosphorylation was also down-regulated by Tim-3 in NK cells, and our experiments demonstrated that NF-κB p65 phosphorylation was enhanced on Tim-3 blockade, consistent with a study of thyroid-associated ophthalmopathy, indicating that Tim-3 suppresses the NFκB p65 pathway to alleviate inflammation [26]. Although we found that Tim-3 could inhibit the ERK and NFκB p65 pathways in NK cells, it failed to suppress IFN-γ production, prompting us to explore whether this involved the NFAT pathway. Our analyses indicate that Tim-3 fails to inhibit NFAT activity in NK cells and, more importantly, that the NFAT pathway has a more significant role in IFN-γ production in NK cells than ERK and NFκB p65 signaling. We interpret these findings as indicating that Tim-3 can act as an inhibitory receptor, suppressing the ERK and NFκB p65 pathways, but fails to inhibit NFAT signaling. Thus the production of IFN-γ in NK cells can be maintained via the NFAT pathway, sustaining the total amount of IFN-γ production (Fig. 7).

This study has some limitations. We included HIV^−^ MSM as control group, HIV^−^ MSM and HIV^+^ MSM had more similar epidemiological and behavioral characteristics compared to HIV^−^ heterosexuals, and thus HIV^−^ MSM were more appropriate to be used as controls, while it might be possible that there were very few HIV-exposed seronegative individuals in HIV^−^ MSM. In this study, IL-12 and IL-15 were used to study the signaling pathway of NK cells for producing IFN-γ, so CD4^+^ T cells were also stimulated with IL-12 and IL-15 to compare with NK cells, while the TCR-mediated CD4^+^ T cell responses were not considered. Although it has been reported that cytokine stimulation is important for CD4^+^ T cell function [27], the TCR-mediated CD4^+^ T cell responses should be further explored in the future.

Overall, our data demonstrate that Tim-3 expression is down-regulated on NK cells during HIV infection, and fails to inhibit IFN-γ production via the NFAT pathway, which is the most significant route for induction of IFN-γ production in NK cells. These findings relating to Tim-3 provide new avenues for further research into the mechanisms underlying HIV infection and immunotherapeutic approaches.

## Supplementary Information

**Additional file 1: Supplementary Figure 1.** The viability of peripheral blood mononuclear cells. Representative flow cytometry plots of the viability of peripheral blood mononuclear cells stimulated with IL-12 and IL-15 for 24 h. All data analyzed using FacsDiva™ 8.0.3 (URL: www.bdbiosciences.com) and FlowJo™ 10.5.0 (URL: www.flowjo.com/flowjo-eula/).

## Data Availability

The datasets generated and/or analyzed during the current study are not publicly available due to privacy restrictions but are available from the corresponding author on reasonable request.

## Abbreviations

HAART: Highly active antiretroviral therapy
HIV: Human immunodeficiency virus
IFN-γ: Interferon-gamma
MSM: Men who have sex with men
NK: Natural killer
PBMCs: Peripheral blood mononuclear cells
