# Supplementary figures and images for: The inhibitory receptor Tim-3 fails to suppress IFN-γ production via the NFAT pathway in NK-cell, unlike that in CD4+ T cells
Source: BMC Immunol. 2021 Apr 9;22:25. doi: 10.1186/s12865-021-00417-9 (PMC8034152; doi:10.1186/s12865-021-00417-9)

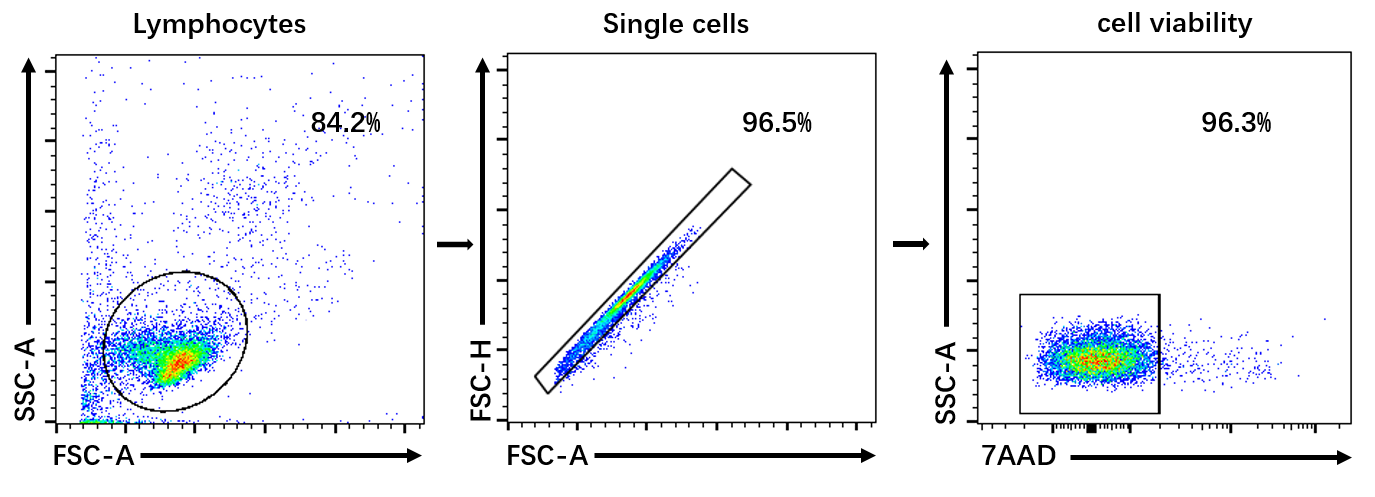

Supplement: Supplementary file 1 — Additional file 1: Supplementary Figure 1. The viability of peripheral blood mononuclear cells. Representative flow cytometry plots of the viability of peripheral blood mononuclear cells stimulated with IL-12 and IL-15 for 24 h. All data analyzed using FacsDiva™ 8.0.3 (URL: www.bdbiosciences.com) and FlowJo™ 10.5.0 (URL: www.flowjo.com/flowjo-eula/). [file 12865_2021_417_MOESM1_ESM.tif]
